# Supplementary material for: A compendium of Amplification-Related Gain Of Sensitivity genes in human cancer
Source: Nat Commun. 2025 Jan 27;16:1077. doi: 10.1038/s41467-025-56301-2 (PMC11772776; doi:10.1038/s41467-025-56301-2)
Supplement: Supplementary file 1 — Supplementary Information [file 41467_2025_56301_MOESM1_ESM.pdf]

# Supplementary Information for "A compendium of Amplification-Related Gain Of Sensitivity (ARGOS) genes in human cancer"


Veronica Rendo[1,2,3,4,5,*], Michael Schubert[6,7,8,9,*], Nicholas Khuu[1,2,4], Maria F. Suarez Peredo Rodriguez[7], Declan Whyte[7], Xiao Ling[7], Anouk van den Brink[7], Kaimeng Huang[4,10], Michelle Swift[10], Yizhou He[4,10], Johanna Zerbib[11], Ross Smith[5], Jonne Raaijmakers[6], Pratiti Bandopadhayay[3,4,12], Lillian M. Guenther[13], Justin H. Hwang[14], Amanda Iniguez[15], Susan Moody[1,3,4], Ji-Heui Seo[1], Elizabeth H. Stover[1,3,4], Levi Garraway[1,4], William C. Hahn[1,3,4], Kimberly Stegmaier[3,4,12], René H. Medema[6], Dipanjan Chowdhury[4,10], Maria Colomé-Tatché[8,16], Uri Ben-David[11,†], Rameen Beroukhim[1,2,3,4,†], and Floris Foijer[7,†]

[1]Department of Medical Oncology and Center for Neuro-Oncology, Dana-Farber Cancer Institute, Boston, MA, USA
[2]Department of Cancer Biology, Dana-Farber Cancer Institute, Boston, MA, USA
[3]Harvard Medical School, Boston, MA, USA
[4]Broad Institute of Harvard and MIT, Cambridge, MA, USA
[5]Department of Immunology, Genetics and Pathology, Uppsala University, Uppsala, Sweden
[6]Oncode Institute, Division of Cell Biology, The Netherlands Cancer Institute, Amsterdam, Netherlands
[7]European Research Institute for the Biology of Ageing, University Medical Center Groningen, Groningen, Netherlands
[8]Institute of Computational Biology, Helmholtz Munich, Neuherberg, Germany
[9]Institute of Bioinformatics, Medical University of Innsbruck, Innsbruck, Austria
[10]Department of Radiation Oncology, Dana-Farber Cancer Institute, Boston, MA, USA
[11]Department of Human Molecular Genetics & Biochemistry, Faculty of Medicine, Tel Aviv University, Tel Aviv, Israel
[12]Department of Pediatrics, Dana-Farber Cancer Institute, Boston, MA, USA
[13]St. Jude Children's Research Hospital, Department of Oncology, Memphis, TN, USA
[14]Division of Hematology, Oncology, and Transplantation, University of Minnesota, Minneapolis, MN, USA
[15]Department of Cancer Biology, Perelman School of Medicine at the University of Pennsylvania, Philadelphia, PA, USA
[16]Biomedical Center (BMC), Physiological Chemistry, Ludwig Maximilians University, Munich, Germany

* These authors contributed equally to this work and are listed alphabetically
† These authors jointly supervised this work and are listed alphabetically

**To whom correspondence should be addressed:**
Veronica Rendo: veronica.rendo@igp.uu.se
Michael Schubert: m.schubert@nki.nl
Uri Ben-David: ubendavid@tauex.tau.ac.il
Rameen Beroukhim: rameen_beroukhim@dfci.harvard.edu
Floris Foijer: f.foijer@umcg.nl

# Supplementary Figures

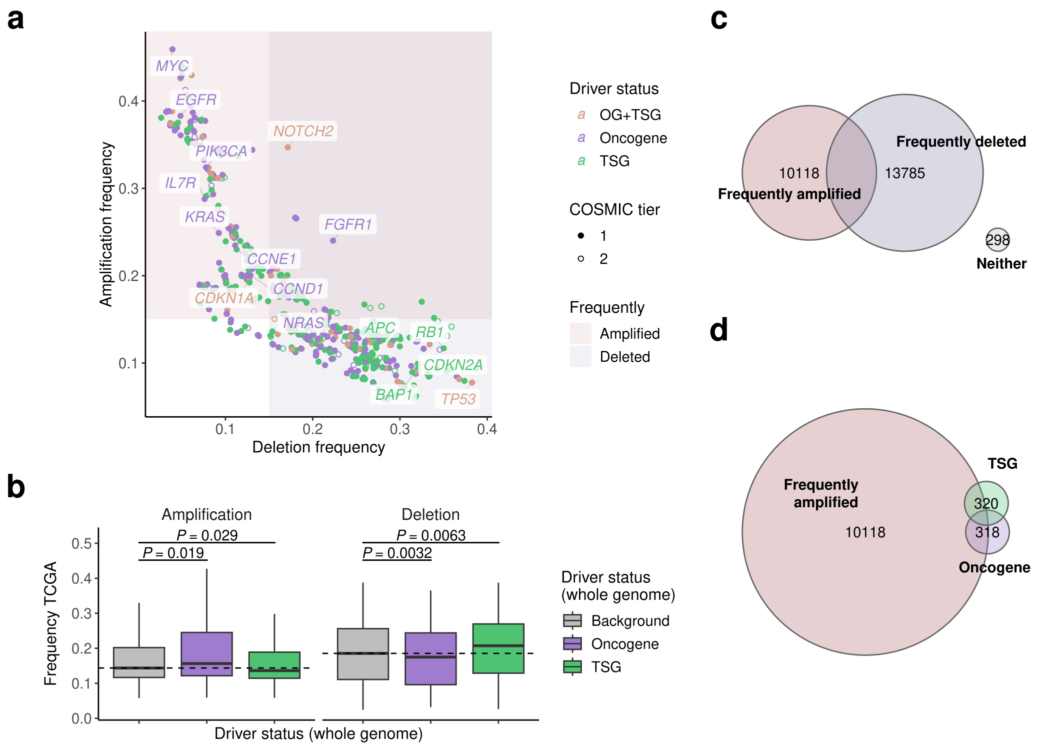


**Figure S1: Landscape of copy number alterations.** **(a)** Frequency of amplifications and deletions in the TCGA with COSMIC driver status highlighted. **(b)** OGs are more frequently amplified, less frequently deleted and TSGs the opposite, **(c)** however most of the cancer genome is frequently amplified or deleted, and **(d)** most amplifications and deletions are not OGs or TSGs. Boxes show median ± quartile, whiskers 1.5x inter-quartile range. *P*-values from two-sided *t*-tests.

**Figure S2: (next page) Compensation analysis.** **(a)** Modeling the TCGA scaling and deviation produced the highest correlation with CCLE when modeling non-cancer cells per tissue (right). **(b)** Gene ontology enrichment of gene deregulation using a linear regression model and the Wald statistic for TCGA (x axis) and CCLE (y axis) shows commonly compensated (negative values) and hyperactivated (positive values) categories. **(c)** Frequently amplified and deleted genes, as well as **(d)** OGs and TSGs are not preferentially compensated. **(e)** Odds ratio of gene class presence within compensated genes. Horizontal lines are 95% confidence intervals. **(f)** Overlap between our compensated genes and previous studies. **(g)** TCGA mutation rates of compensated genes of different studies. **(h-i)** Identified compensated genes are also expressed less than expected in RPE-1 clones. Fold changes were calculated over the parental RPE-1 line and then normalized per amplified chromosome. **(j)** Overlap between compensated genes and genes previously identified as disease-causing when amplified (Wilcox test). **(k)** Compensation scores of common and dataset-specific genes (horizontal panels) in their respective datasets (vertical panels), as well as **(l)** enrichment of genes found in three or more analyses (Fisher's Exact Test). Compensated genes in (k) are shown in larger squares relative to those not passing our threshold criteria. Boxes show median ± quartile, whiskers 1.5x inter-quartile range. *P*-values from a linear regression model (a, b) two-sided *t*-tests (c, d, g, i, j), one-sided *t*-tests (h) and Fisher's Exact Tests (e, l).

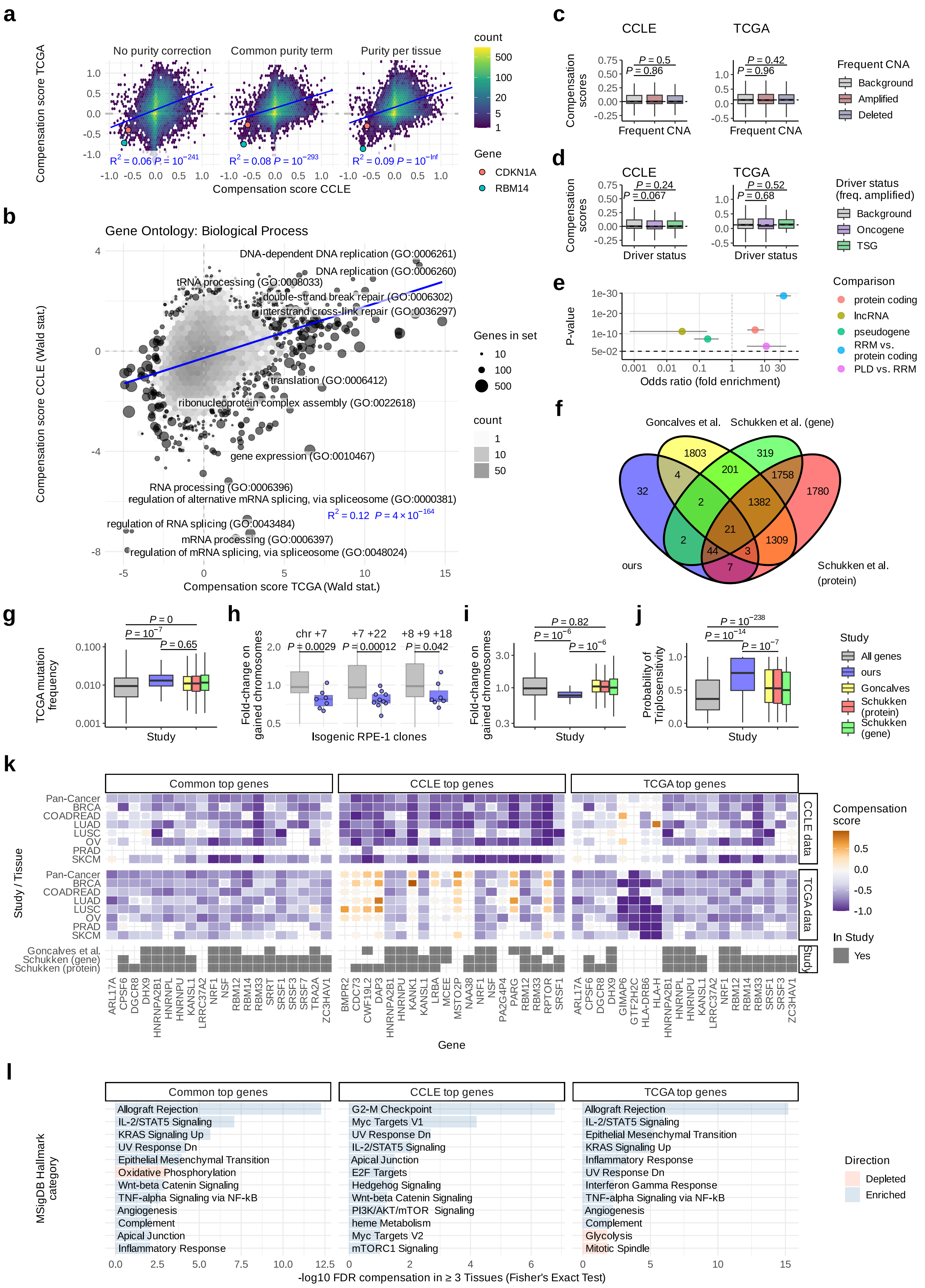
a
No purity correction
Common purity term
Purity per tissue
Compensation score TCGA
Compensation score CCLE
count
500
100
20
5
1
Gene
CDKN1A
RBM14
$R^2$ = 0.06 $P$ = $10^{-241}$
$R^2$ = 0.08 $P$ = $10^{-293}$
$R^2$ = 0.09 $P$ = $10^{-Inf}$
b
Gene Ontology: Biological Process
DNA-dependent DNA replication (GO:0006261)
DNA replication (GO:0006260)
tRNA processing (GO:0008033)
double-strand break repair (GO:0006302)
interstrand cross-link repair (GO:0036297)
translation (GO:0006412)
ribonucleoprotein complex assembly (GO:0022618)
gene expression (GO:0010467)
RNA processing (GO:0006396)
regulation of alternative mRNA splicing, via spliceosome (GO:0000381)
$R^2$ = 0.12 $P$ = 4 × $10^{-164}$
regulation of RNA splicing (GO:0043484)
mRNA processing (GO:0006397)
regulation of mRNA splicing, via spliceosome (GO:0048024)
Compensation score CCLE (Wald stat.)
Compensation score TCGA (Wald stat.)
Genes in set
10
100
500
count
1
10
50
c
CCLE
TCGA
Compensation scores
$P$ = 0.5
$P$ = 0.86
$P$ = 0.42
$P$ = 0.96
Frequent CNA
Background
Amplified
Deleted
d
CCLE
TCGA
$P$ = 0.24
$P$ = 0.067
$P$ = 0.52
$P$ = 0.68
Driver status
Driver status (freq. amplified)
Background
Oncogene
TSG
e
P-value
Odds ratio (fold enrichment)
Comparison
protein coding
lncRNA
pseudogene
RRM vs. protein coding
PLD vs. RRM
f
Goncalves et al.
Schukken et al. (gene)
Schukken et al. (protein)
ours
1803
319
4
201
1758
32
1780
2
1382
21
2
1309
44
3
7
g
TCGA mutation frequency
$P$ = 0
$P$ = $10^{-7}$
$P$ = 0.65
Study
h
Fold-change on gained chromosomes
chr +7
+7 +22
+8 +9 +18
$P$ = 0.0029
$P$ = 0.00012
$P$ = 0.042
Isogenic RPE-1 clones
i
Fold-change on gained chromosomes
$P$ = 0.82
$P$ = $10^{-6}$
$P$ = $10^{-6}$
Study
j
Probability of Triplosensitivity
$P$ = $10^{-238}$
$P$ = $10^{-14}$
$P$ = $10^{-7}$
Study
Study
All genes
ours
Goncalves
Schukken (protein)
Schukken (gene)
k
Common top genes
CCLE top genes
TCGA top genes
Study / Tissue
Pan-Cancer
BRCA
COADREAD
LUAD
LUSC
OV
PRAD
SKCM
Goncalves et al.
Schukken (gene)
Schukken (protein)
CCLE data
TCGA data
Study
Compensation score
In Study
Yes
Gene
l
Common top genes
Allograft Rejection
IL-2/STAT5 Signaling
KRAS Signaling Up
UV Response Dn
Epithelial Mesenchymal Transition
Oxidative Phosphorylation
Wnt-beta Catenin Signaling
TNF-alpha Signaling via NF-kB
Angiogenesis
Complement
Apical Junction
Inflammatory Response
CCLE top genes
G2-M Checkpoint
Myc Targets V1
UV Response Dn
IL-2/STAT5 Signaling
Apical Junction
E2F Targets
Hedgehog Signaling
Wnt-beta Catenin Signaling
PI3K/AKT/mTOR Signaling
heme Metabolism
Myc Targets V2
mTORC1 Signaling
TCGA top genes
Allograft Rejection
IL-2/STAT5 Signaling
Epithelial Mesenchymal Transition
KRAS Signaling Up
Inflammatory Response
UV Response Dn
Interferon Gamma Response
TNF-alpha Signaling via NF-kB
Angiogenesis
Complement
Glycolysis
Mitotic Spindle
MSigDB Hallmark category
-log10 FDR compensation in ≥ 3 Tissues (Fisher's Exact Test)
Direction
Depleted
Enriched

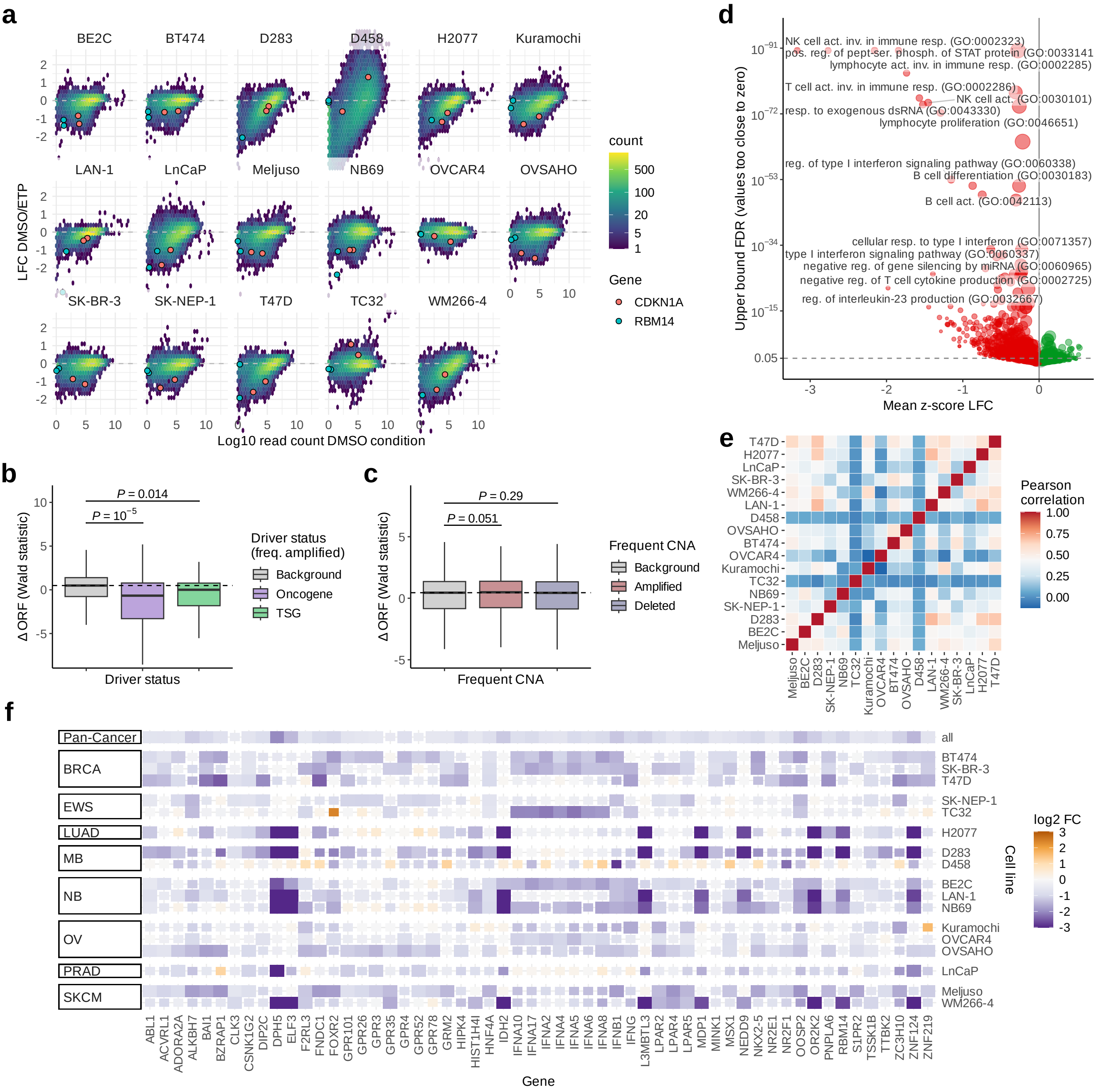


**Figure S3: ORF screen analysis.** **(a)** Overview of the expression (x axis) and dropout levels (y axis) for all screens with two genes of interest highlighted. Most genes are represented by two ORFs in the library. **(b)** Both OGs and TSGs dropped out preferentially in the ORF screens over other genes. **(c)** We observed no differential ORF dropout of frequently amplified or deleted genes. **(d)** Volcano plot of Gene Ontology categories that are preferentially enriched (green, positive values) or drop out (red, negative values) across ORF screens. Boxes show median ± quartile, whiskers 1.5x inter-quartile range. *P*-values from two-sided *t*-tests. **(e)** Pearson correlation of log fold-changes across screens shows values of 0.4 to 0.6, with three less correlated screens. We did not observe a trend where more similar tissues showed a higher correlation and hence quantified viability difference common to all screens using a linear regression model. **(f)** Gene dropout (*log2* fold changes) in the pan-cancer, as well as in cell-line specific analyses. Genes common to four or more ORF screens are shown, with hits passing our thresholds indicated in larger square size.

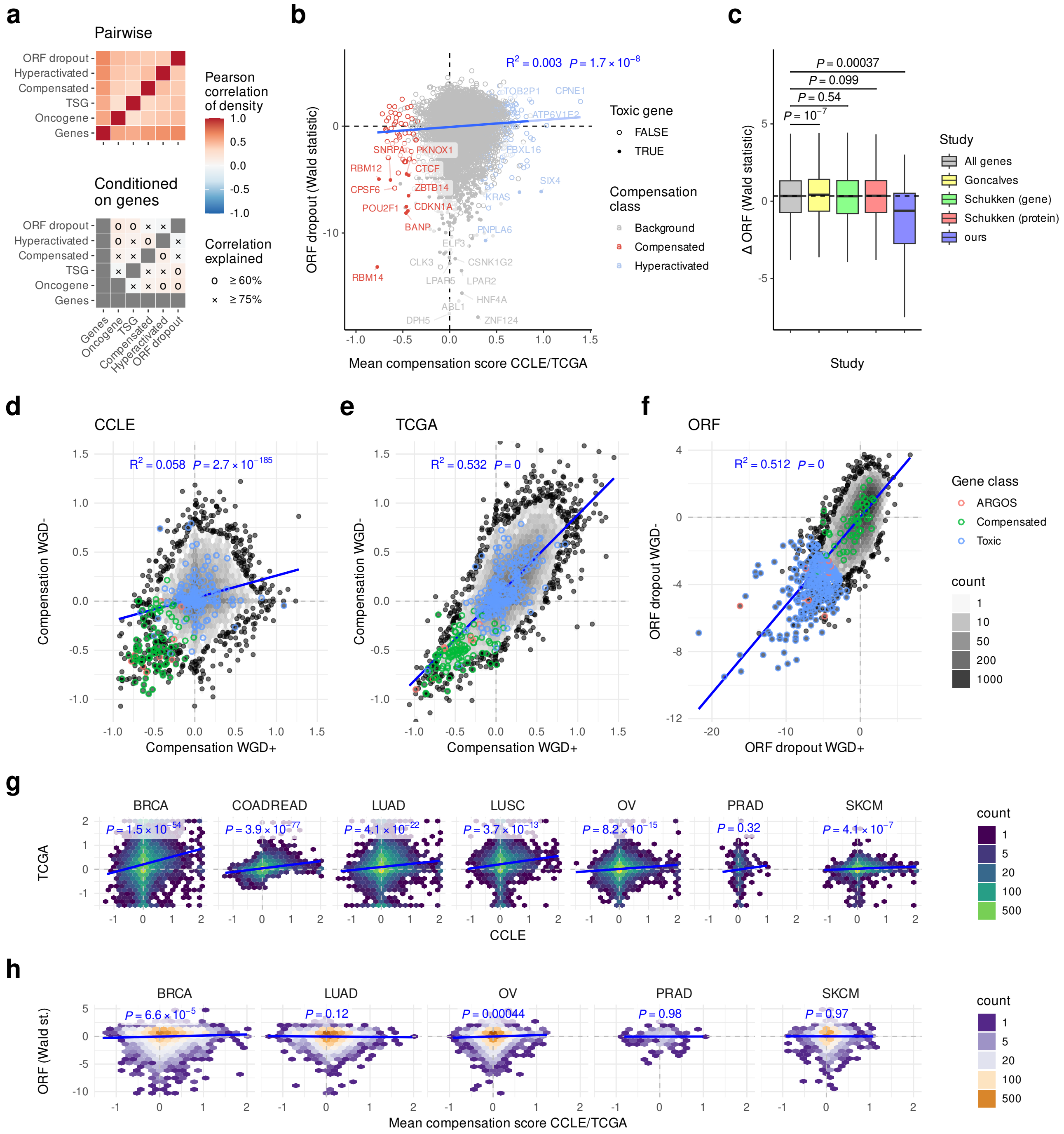


**Figure S4: Characterization of compensation and ORF overlap.** **(a)** Pearson correlation of the gene densities in Fig. 3a (top) and adjusted for gene densities (bottom) shows that gene density along the genome explains most of the observed correlations. **(b)** Compensation vs. ORF dropout plot with genes in the respective compensation categories highlighted. **(c)** ORF dropout for compensated gene sets from different studies ($t$ test). **(d-f)** Comparison of compensation scores in CCLE (d) and TCGA (e), as well as ORF toxicity (f) between samples with genome doubling and without. **(g)** Correlation between compensation scores for CCLE and TCGA in different cancer types. **(h)** Comparison of compensation and toxicity in different cancer types. Boxes show median ± quartile, whiskers 1.5x inter-quartile range. $R^2$ and $P$-values from linear regression models.

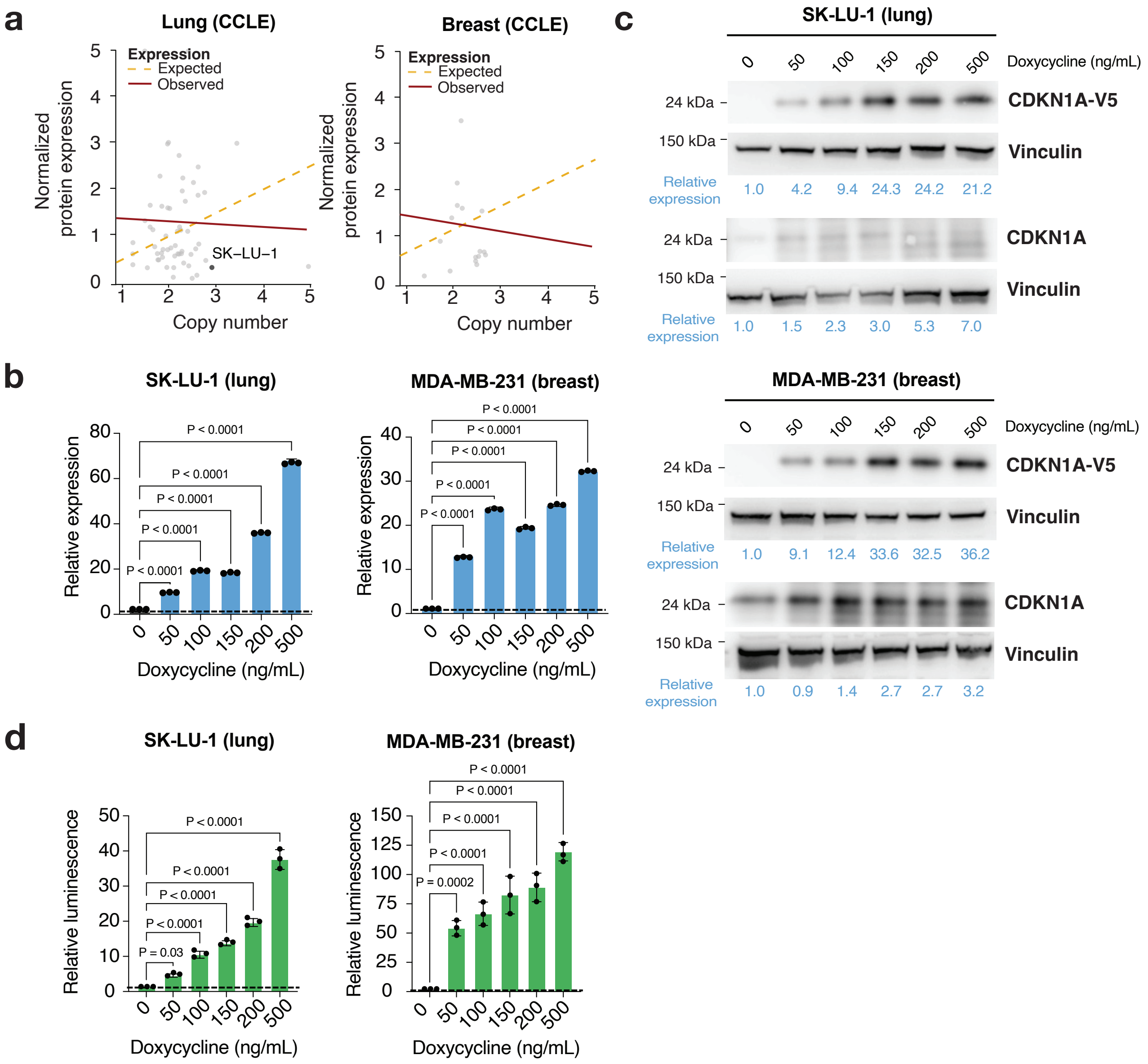


**Figure S5: Generation of doxycycline-inducible cell model systems to study CDKN1A overexpression.** **(a)** CDKN1A protein expression level and copy number for lung and breast cancer cell lines in CCLE. No data for MDA-MB-231 cells available. Expected (yellow) and observed (red) levels of CDKN1A protein expression are shown for each tumor type. **(b)** Increasing levels of *CDKN1A* transcript were detected by RT-qPCR in SK-LU-1$^{\mathrm{CDKN1A}}$ and MDA-MB-231$^{\mathrm{CDKN1A}}$ cells when treated with increasing concentrations of doxycycline. Expression values are shown relative to untreated (0 ng/mL) control. Mean and standard deviation of three technical replicates. Data analyzed with one-way ANOVA. **(c)** Increases in exogenous (V5-tagged) and total CDKN1A protein were detected by immunoblotting in the same cell models. Vinculin was used as loading control. Expression values are shown relative to untreated (0 ng/mL) control. **(d)** Activity of luciferase cells was determined by a reporter assay. Increases in luminescence were detected for both cell lines at increasing concentrations of doxycycline. Luminescence values are shown relative to untreated (0 ng/mL) control. Mean and standard deviation of three replicates. Data analyzed with one-way ANOVA. Source data for Figures S5b-d are provided as Source Data files.

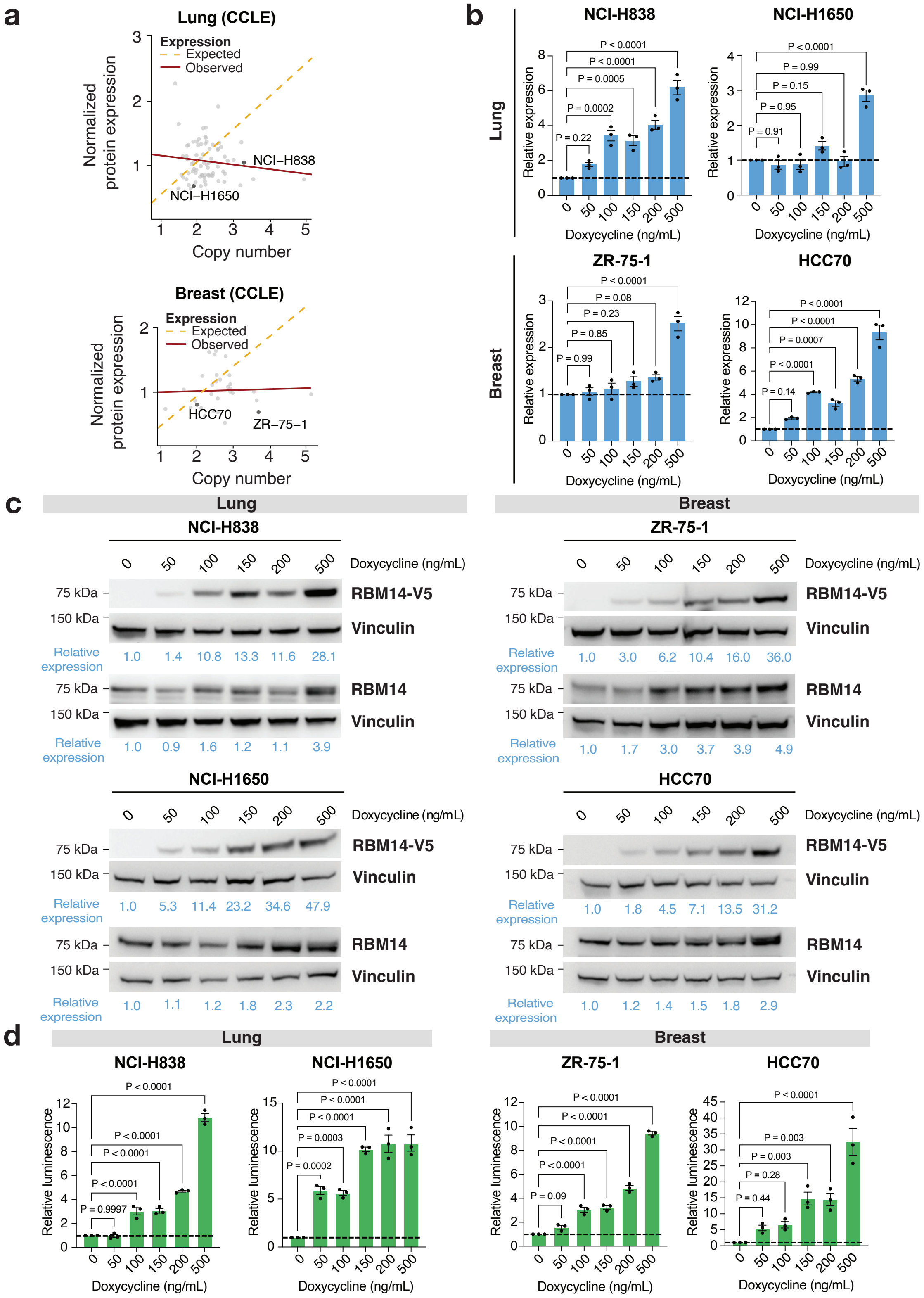


**Figure S6: Generation of doxycycline-inducible cell model systems to study RBM14 overexpression.** **(a)** Cell lines chosen for functional validation show similar compensation profiles based on protein expression level and copy number. **(b)** Increasing levels of RBM14 transcript were detected by RT-qPCR in NCI-H838$^{RBM14}$, NCI-H1650$^{RBM14}$, ZR-75-1$^{RBM14}$ and HCC70$^{RBM14}$ cell lines when treated with increasing concentrations of doxycycline. Expression values are shown relative to untreated (0 ng/mL) control. Mean and standard deviation of three technical replicates. Data analyzed with one-way ANOVA. **(c)** Increases in exogenous (V5-tagged) and total RBM14 protein were detected by immunoblotting in all four cell models. Vinculin was used as loading control. Expression values are shown relative to untreated (0 ng/mL) control. **(d)** Activity of luciferase cells was determined by a reporter assay. Increases in luminescence were detected for both cell lines at increasing concentrations of doxycycline. Luminescence values are shown relative to untreated (0 ng/mL) control. Mean and standard deviation of three replicates. Data analyzed with one-way ANOVA. Source data for Figures S6b-d are provided as Source Data files.

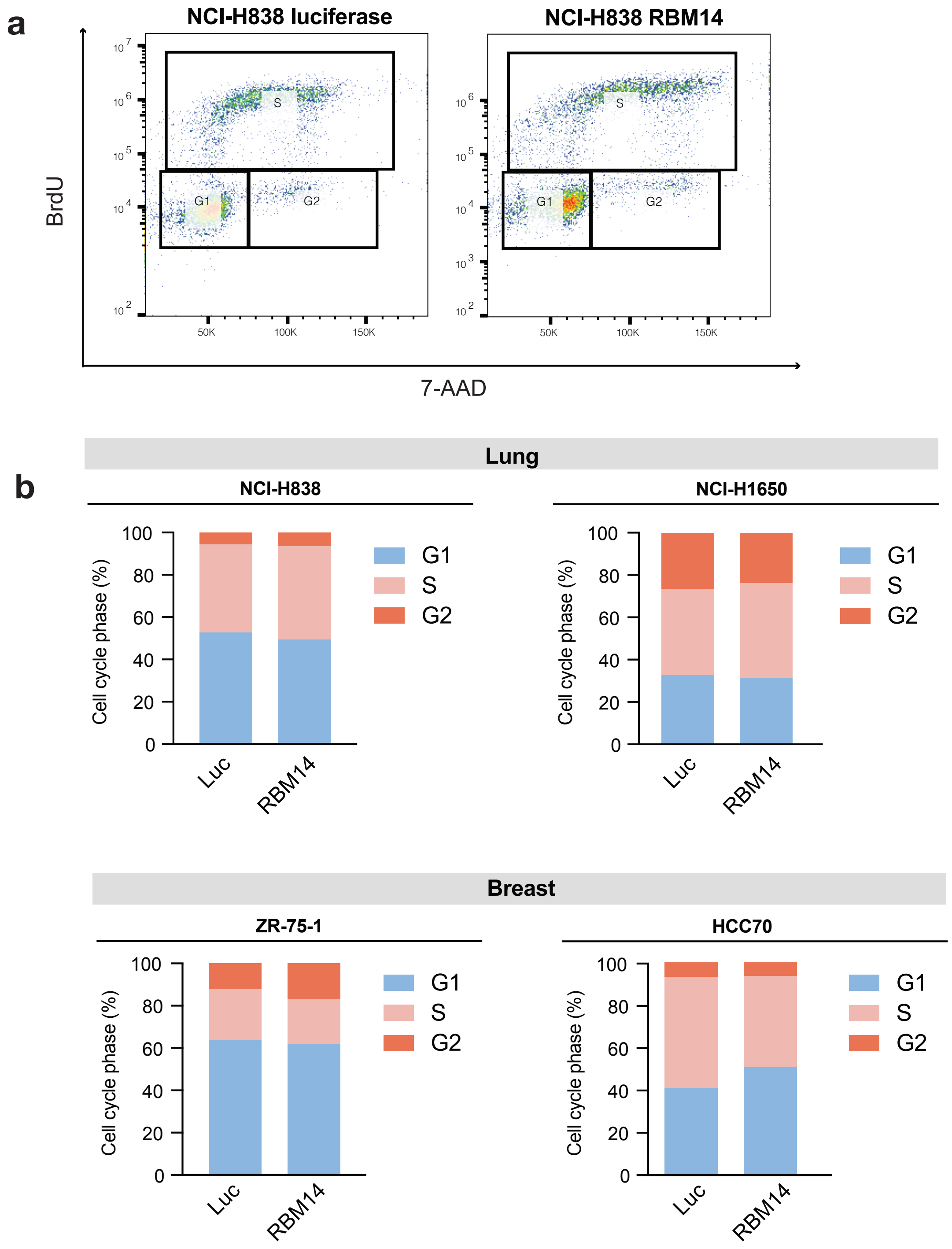


**Figure S7: Cell cycle analysis in compensated cell line models of RBM14 overexpression.** **(a)** Representative image of a BrdU incorporation assay in NCI-H838 cells overexpressing RBM14 or luciferase control (of two biological replicates). In this assay, 7-AAD was used as nucleic acid staining to quantify total DNA content. Source data are provided as a Source Data file. **(b)** Percentage of cells in each phase of the cell cycle following RBM14 or luciferase overexpression in lung (NCI-H838, NCI-H1650) and breast (ZR-75-1, HCC70) cancer cell lines (in three replicates, each with 10,000 recorded events).

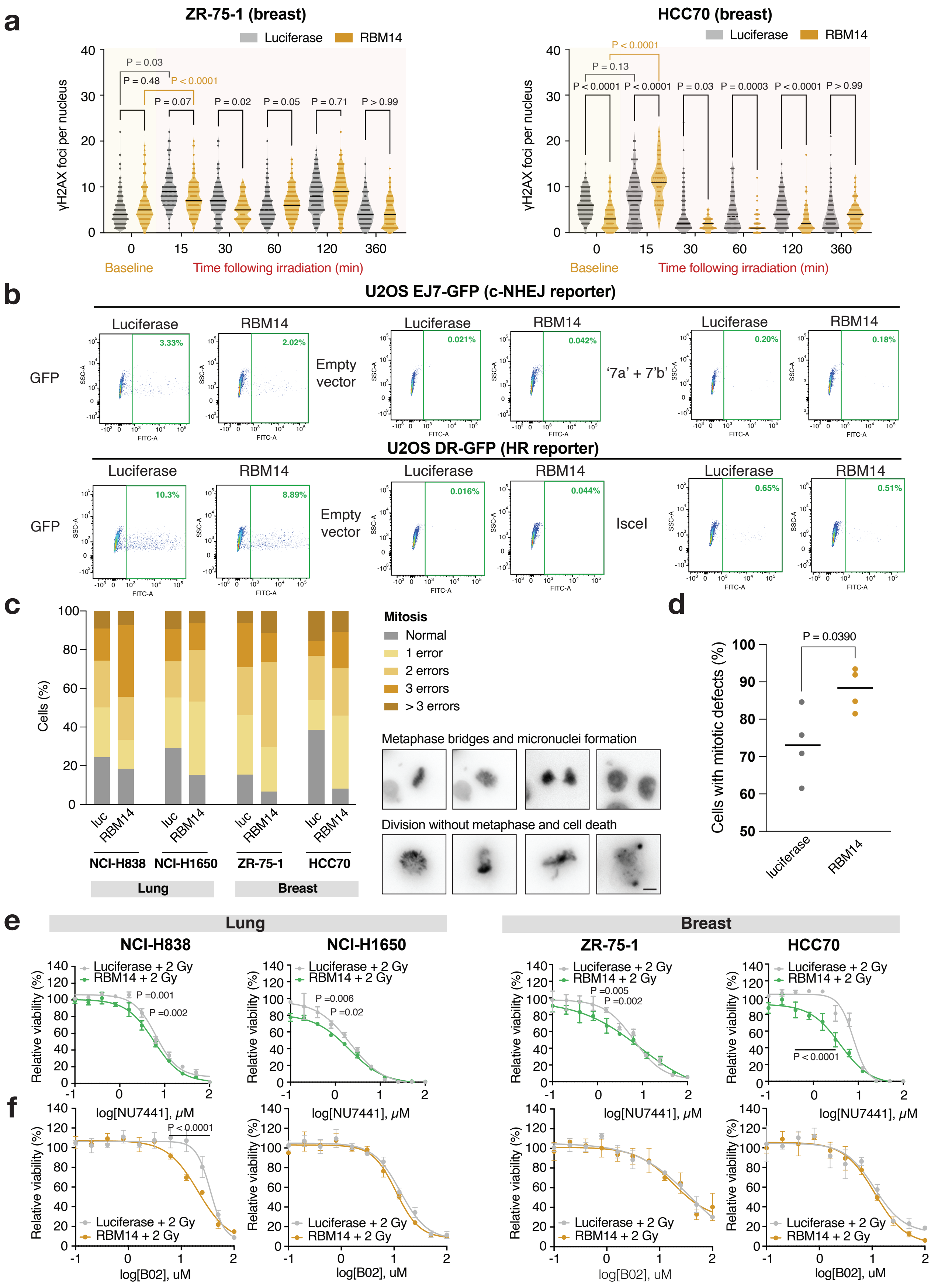
a
ZR-75-1 (breast)
Luciferase
RBM14
γH2AX foci per nucleus
P = 0.03
P = 0.48
P < 0.0001
P = 0.07
P = 0.02
P = 0.05
P = 0.71
P > 0.99
Baseline
Time following irradiation (min)
HCC70 (breast)
P < 0.0001
P = 0.13
P < 0.0001
P < 0.0001
P = 0.03
P = 0.0003
P < 0.0001
P > 0.99
b
U2OS EJ7-GFP (c-NHEJ reporter)
GFP
Empty vector
'7a' + 7'b'
3.33%
2.02%
0.021%
0.042%
0.20%
0.18%
U2OS DR-GFP (HR reporter)
IsceI
10.3%
8.89%
0.016%
0.044%
0.65%
0.51%
SSC-A
FITC-A
c
Cells (%)
Mitosis
Normal
1 error
2 errors
3 errors
> 3 errors
luc
RBM14
NCI-H838
NCI-H1650
ZR-75-1
HCC70
Lung
Breast
Metaphase bridges and micronuclei formation
Division without metaphase and cell death
d
Cells with mitotic defects (%)
P = 0.0390
luciferase
RBM14
e
Lung
Breast
NCI-H838
NCI-H1650
ZR-75-1
HCC70
Luciferase + 2 Gy
RBM14 + 2 Gy
Relative viability (%)
P =0.001
P =0.002
P =0.006
P =0.02
P =0.005
P =0.002
P < 0.0001
log[NU7441], μM
f
P < 0.0001
log[B02], uM

**Figure S8: (previous page) DNA damage response in cell line models of RBM14 overexpression. (a)** The number of yH2AX foci was quantified by immunofluorescence in breast ZR-75-1$^{\mathrm{RBM14/luc}}$ and HCC70$^{\mathrm{RBM14/luc}}$ cells following 2 Gy ionizing radiation (IR). Data analyzed by two-way ANOVA. The horizontal bar in each violin plot indicates the mean. Images analyzed from two independent biological replicates. **(b)** Representative images for each condition of the DNA damage reporter assay described in Fig. 6c. The fraction of GFP-positive cells are shown in green rectangles. **(c)** Quantification of the number of mitotic defects per cell after 2 Gy IR. Representative images of observed mitotic alterations, including DNA bridges, micronuclei formation and cell division without metaphase (images on the right panel). Scale bar: 12 µM. Data from two biological replicates **(d)** Rates of cells with mitotic defects following DNA damage by 2 Gy IR. The mean of each condition is shown in black. Data analyzed with unpaired two-tailed t-test. **(e-f)** Response of lung and breast cell line models to (e) DNA-PK inhibition (NU7441) and (f) RAD51 (B02) inhibition. Cells were irradiated with 2 Gy and treated with each small-molecule for 72h. Cell viability relative to DMSO control was assessed in a CellTiterGlo luminescence assay. Data analyzed with two-way ANOVA. Source data for Figures S8a and S8d-f are provided as Source Data files.

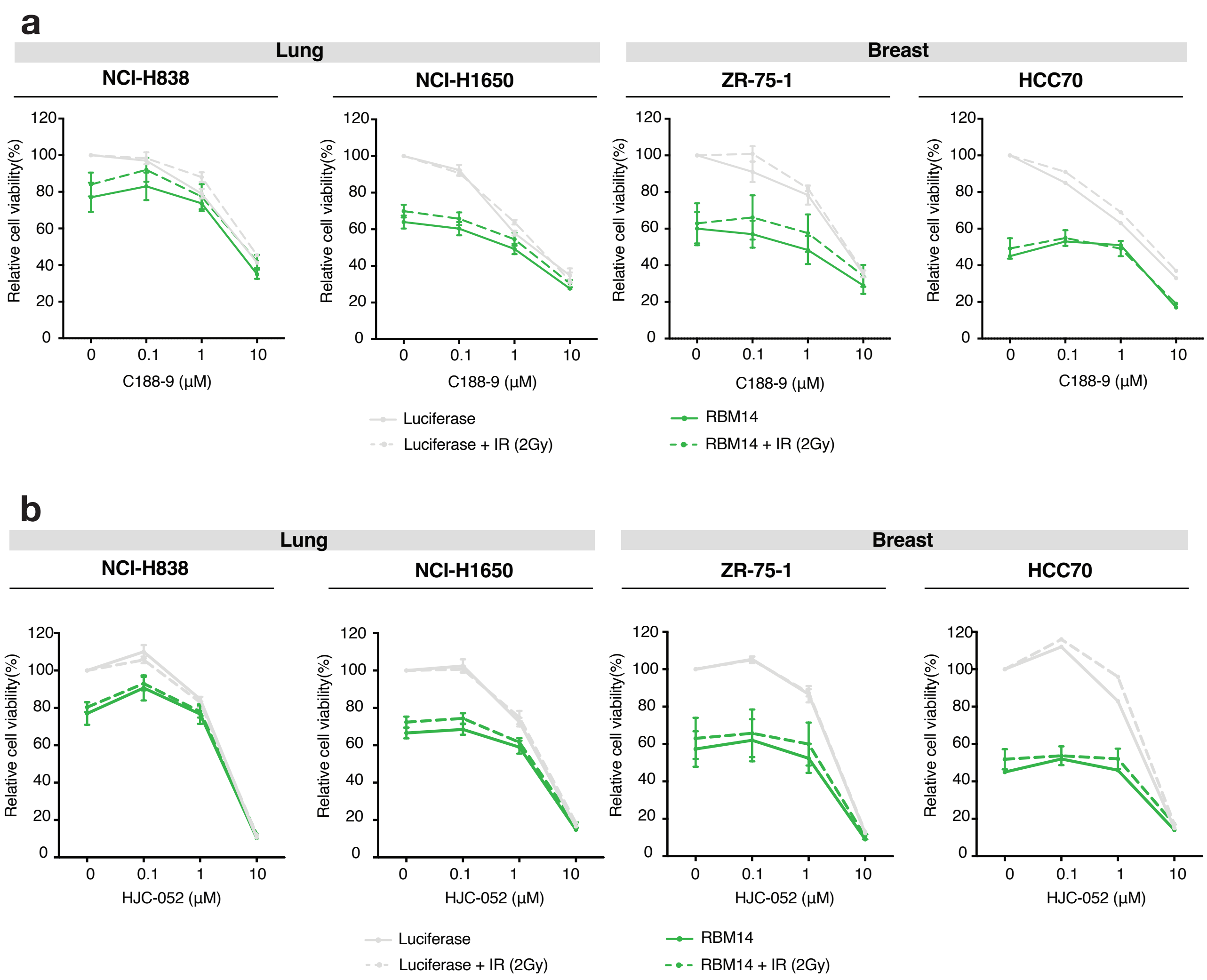


**Figure S9: RBM14 overexpression modulates response to STAT3 inhibition. (a-b)** Response of lung and breast cell line models to increasing concentrations of the STAT3 inhibitors (a) C188-9 and (b) HJC052. Cells were irradiated with 2 Gy and treated with each small-molecule for 72h. Cell viability relative to DMSO control was assessed in a MTT absorbance assay. Source data for Figures S9a and S9b are provided as Source Data files.
