# Supplementary material for: A compendium of Amplification-Related Gain Of Sensitivity genes in human cancer
Source: Nat Commun. 2025 Jan 27;16:1077. doi: 10.1038/s41467-025-56301-2 (PMC11772776; doi:10.1038/s41467-025-56301-2)
Supplement: Supplementary file 3 — Description of Additional Supplementary Files [file 41467_2025_56301_MOESM3_ESM.pdf]

Description of Additional Supplementary Files

Supplementary Data 1: Gene lists for further exploration

In this file, we provide our identified pan-cancer gene sets as lists that can easily be copied to other platforms (*e.g.* https://maayanlab.cloud/Enrichr/) for further exploration. They contain only gene symbols present in these lists.

Supplementary Data 2: CCLE gene expression compensation

Results of the Negative Binomial gene expression compensation using CCLE data. Different CCLE cancer types in sheets, *Pan-Cancer* refers to the inclusion of all cancer types, *panWGD+* and *panWGD-* to their respective genome doubling subset. The column *estimate* is the mean, *std.error* the standard deviation of the posterior, relative to *eup_reads*. The normalized read count assigned to the scaling term is called *eup_reads*. The *z-score* is the number of standard deviations of the posterior from the origin, which serves as a basis for *p-value* calculation, where *adj.p* is corrected by False Discovery Rate. The final *compensation* column is the shrunk estimate. *Rhat* and *n_eff* are the identically named convergence measures. The column *type* refers to whether this gene passed our cutoffs to be *Compensated* or *Hyperactivated*.

Supplementary Data 3: TCGA gene expression compensation

Results of the Negative Binomial gene expression compensation using TCGA data. Different TCGA cancer types in sheets, *Pan-Cancer* refers to the inclusion of all cancer types, *panWGD+* and *panWGD-* to their respective genome doubling subset. The column *estimate* is the mean, *std.error* the standard deviation of the posterior, relative to *eup_reads*. The normalized read count assigned to the scaling term is called *eup_reads*, to non-cancer cells *stroma_reads*. The *z-score* is the number of standard deviations of the posterior from the origin, which serves as a basis for *p-value* calculation, where *adj.p* is corrected by False Discovery Rate. The final *compensation* column is the shrunk estimate. *Rhat* and *n_eff* are the identically named convergence measures. The column *type* refers to whether this gene passed our cutoffs to be *Compensated* or *Hyperactivated*.

Supplementary Data 4: Assembled ORF screen data

Source data of the different ORF screens incorporated in this study. The first two sheets are each a collection of screen results, where their columns refer to the cell line used and whether this is an early time point (ETP), late time point (DMSO), or the change between (LFC). Plasmid sizes are listed in sheet 3, and which cell line belongs to which library in sheet 4.

Supplementary Data 5: ORF toxicity associations

Results of the linear regression model for all ORF screens (*Pan-Cancer*) or each individual cell line. The columns *estimate*, *std.error*, *statistic,* and *p.value* are default outputs from a linear regression model in R, with *adj.p* added for False Discovery Rate correction of p-values. The column *size* refers to how many ORFs were included for that gene, *is_toxic* whether this gene passed our thresholds.
