# Supplementary material for: A compendium of Amplification-Related Gain Of Sensitivity genes in human cancer
Source: Nat Commun. 2025 Jan 27;16:1077. doi: 10.1038/s41467-025-56301-2 (PMC11772776; doi:10.1038/s41467-025-56301-2)
Supplement: Supplementary file 9 — Reporting Summary [file 41467_2025_56301_MOESM9_ESM.pdf]

nature portfolio

Corresponding author(s): Veronica Rendo, Michael Schubert, Uri Ben-David, Rameen Beroukhim, Floris Foijer

Last updated by author(s): Nov 16, 2024

Reporting Summary

Nature Portfolio wishes to improve the reproducibility of the work that we publish. This form provides structure for consistency and transparency in reporting. For further information on Nature Portfolio policies, see our Editorial Policies and the Editorial Policy Checklist.

Statistics

For all statistical analyses, confirm that the following items are present in the figure legend, table legend, main text, or Methods section.

n/a	Confirmed	
☐	☒	The exact sample size (n) for each experimental group/condition, given as a discrete number and unit of measurement
☐	☒	A statement on whether measurements were taken from distinct samples or whether the same sample was measured repeatedly
☐	☒	The statistical test(s) used AND whether they are one- or two-sided<br>*Only common tests should be described solely by name; describe more complex techniques in the Methods section.*
☐	☒	A description of all covariates tested
☐	☒	A description of any assumptions or corrections, such as tests of normality and adjustment for multiple comparisons
☐	☒	A full description of the statistical parameters including central tendency (e.g. means) or other basic estimates (e.g. regression coefficient) AND variation (e.g. standard deviation) or associated estimates of uncertainty (e.g. confidence intervals)
☐	☒	For null hypothesis testing, the test statistic (e.g. F, t, r) with confidence intervals, effect sizes, degrees of freedom and P value noted<br>*Give P values as exact values whenever suitable.*
☐	☒	For Bayesian analysis, information on the choice of priors and Markov chain Monte Carlo settings
☐	☒	For hierarchical and complex designs, identification of the appropriate level for tests and full reporting of outcomes
☐	☒	Estimates of effect sizes (e.g. Cohen's d, Pearson's r), indicating how they were calculated

Our web collection on statistics for biologists contains articles on many of the points above.

Software and code

Policy information about availability of computer code

Data collection	Public data was re-analyzed and data availability is stated in the manuscript.
Data analysis	All analysis code, as well as a list of used packages and their versions, is available at https://github.com/mschubert/ToxicGenes.

For manuscripts utilizing custom algorithms or software that are central to the research but not yet described in published literature, software must be made available to editors and reviewers. We strongly encourage code deposition in a community repository (e.g. GitHub). See the Nature Portfolio guidelines for submitting code & software for further information.

Data

Policy information about availability of data

All manuscripts must include a data availability statement. This statement should provide the following information, where applicable:

- Accession codes, unique identifiers, or web links for publicly available datasets
- A description of any restrictions on data availability
- For clinical datasets or third party data, please ensure that the statement adheres to our policy

CCLE data was downloaded from DepMap (https://depmap.org/portal/data_page/?tab=allData) 73, including cell line annotations ("Cell_lines_annotations_20181226.txt"), RNA-seq gene counts ("CCLE_RNAseq_genes_counts_20180929.gct.gz"), log2 copy number changes over the mean per gene ("CCLE_copynumber_byGene_2013-12-03.txt.gz"), and WGD ("OmicsSignatures.csv"). TCGA copy number call significance was obtained by GISTIC copy

number calls from the Broad TCGA copy number portal (tumorscape.org) using the Tumorscape 1.2.1 analysis data ("2015-06-01 stddata__2015_04_02 arm-level peel-off", available from the original authors on request) 1,75. Tumor purity estimates were used from the ESTIMATE algorithm 76 applied to the extended TCGA cohorts (https://static-content.springer.com/esm/art%3A10.1038%2Fncomms9971/MediaObjects/41467_2015_BFncomms9971_MOESM1236_ESM.xlsx) 77. Oncogenes and Tumor Suppressor gene lists were downloaded from the COSMIC gene census ("Census_COSMIC96.tsv", requires an account at https://cancer.sanger.ac.uk/) 38 and included in the respective lists if they were listed as Hallmark, Tier 1, or Tier 2. Individual copy number events were extracted from previously published Ziggurat Deconstruction analysis of copy number states ("TCGA.all_cancers.150601.zigg_events.160923.txt", available from the original authors on request) 75. WGD information was downloaded from the TCGA Pan-Cancer Atlas (https://api.gdc.cancer.gov/data/4f277128-f793-4354-a13d-30cc7fe9f6b5). For patient survival analyses, we used recently published data from a colorectal cancer cohort ("Supplementary_Table_01.xlsx" at https://static-content.springer.com/esm/art%3A10.1038%2Fs41586-024-07769-3/MediaObjects/41586_2024_7769_MOESM3_ESM.zip) 66. Other source data are provided with this paper.

Research involving human participants, their data, or biological material

Policy information about studies with human participants or human data. See also policy information about sex, gender (identity/presentation), and sexual orientation and race, ethnicity and racism.

Reporting on sex and gender	We do not include reporting on sex and gender in the study.
Reporting on race, ethnicity, or other socially relevant groupings	We do not include reporting on race, ethnicity, or other socially relevant groupings in the study.
Population characteristics	We do not include population characteristics in the study.
Recruitment	No patients were recruited for this study.
Ethics oversight	No ethical protocols were required for the execution of this study.

Note that full information on the approval of the study protocol must also be provided in the manuscript.

Field-specific reporting

Please select the one below that is the best fit for your research. If you are not sure, read the appropriate sections before making your selection.

☒ Life sciences ☐ Behavioural & social sciences ☐ Ecological, evolutionary & environmental sciences

For a reference copy of the document with all sections, see nature.com/documents/nr-reporting-summary-flat.pdf

Life sciences study design

All studies must disclose on these points even when the disclosure is negative.

Sample size	Functional validation experiments were conducted in as many pairs of breast and lung cancer cell lines that we could obtain based on CDKN1A and RBM14 compensation profiles.
Data exclusions	No data points were excluded from the analyses.
Replication	Growth curves were performed in three technical replicates per biological sample and timepoint. Nascent protein synthesis assays were conducted by analysis of five independent image frames. Flow cytometry assays were conducted in three technical replicates per biological sample and run with negative and positive controls. RT-qPCR and luciferase reporter assays were conducted in three technical replicates per biological sample. Quantification of DNA damage was conducted in at least two biological replicates, each containing at least 30 independent cells for each condition and each timepoint. DNA reporter assays were conducted in three technical replicates per biological sample. Immunoblots were performed in at least two biological replicates. Drug response curves were conducted in three technical replicates per biological sample at each concentration. Quantification of STING area was conducted in at least 9 independent image frames per condition. Co-culture experiments with NK-92 cells were conducted in three biological replicates. All reported experimental findings were reliably reproduced.
Randomization	No randomization was used.
Blinding	No blinding was used.

Reporting for specific materials, systems and methods

We require information from authors about some types of materials, experimental systems and methods used in many studies. Here, indicate whether each material, system or method listed is relevant to your study. If you are not sure if a list item applies to your research, read the appropriate section before selecting a response.

Materials & experimental systems

n/a	Involved in the study
☐	☒ Antibodies
☐	☒ Eukaryotic cell lines
☒	☐ Palaeontology and archaeology
☒	☐ Animals and other organisms
☒	☐ Clinical data
☒	☐ Dual use research of concern
☒	☐ Plants

Methods

n/a	Involved in the study
☒	☐ ChIP-seq
☐	☒ Flow cytometry
☒	☐ MRI-based neuroimaging

Antibodies

Antibodies used

*Primary antibodies:
rabbit V5 at 1:1,000 dilution (cat. no. 13202S, Cell Signaling Technology)
rabbit CDKN1A at 1:1,000 dilution (cat. no. 2947S, Cell Signaling Technology)
rabbit RBM14 at 1:1,000 dilution (cat. no. Ab70636, Abcam)
rabbit DNA-PKcs S2056 at 1:1,000 dilution (cat. no. Ab124918, Abcam)
mouse vinculin at 1:1,000 dilution (cat. no. V9131, Millipore Sigma)
mouse γH2A.X at 1:3,000 dilution (cat. no. 05-636, Millipore)
mouse STING at 1:100 dilution (cat. no. PA5-23381, Invitrogen)

*Secondary antibodies:
goat anti-rabbit at 1:3,000 dilution (cat. no. 7074S, ThermoFisher)
goat anti-mouse at 1:10,000 dilution (cat. no. 7076S, ThermoFisher)

Validation

Antibodies were selected based on their use in the literature in human cancer cell lines, and previous experience of the authors. Full antibody information is provided in the Methods section of the paper. Positive and negative controls were used in all experiments including antibodies.

*Product citations (n) for primary antibodies:
rabbit V5 (353): https://www.cellsignal.com/products/primary-antibodies/v5-tag-d3h8q-rabbit-mab/13202
rabbit CDKN1A (1982): https://www.cellsignal.com/products/primary-antibodies/p21-waf1-cip1-12d1-rabbit-mab/2947
rabbit RBM14 (2): https://www.abcam.com/en-se/products/primary-antibodies/rbm14-antibody-ab70636#wb
rabbit DNA-PKcs S2056 (20): https://www.abcam.com/en-us/products/primary-antibodies/dna-pkcs-phospho-s2056-antibody-epr5670-ab124918
mouse vinculin (1812): https://www.sigmaaldrich.com/SE/en/product/sigma/v9131
mouse γH2A.X (10): https://www.merckmillipore.com/SE/en/product/Anti-phospho-Histone-H2A.X-Ser139-Antibody-clone-JBW301,MM_NF-05-636
mouse STING (2): https://www.thermofisher.com/antibody/product/STING-Antibody-Polyclonal/PA5-23381

Eukaryotic cell lines

Policy information about cell lines and Sex and Gender in Research

Cell line source(s)

NCI-H838 (cat. no. CRL-5844; ATCC)
NCI-H1650 (cat. no. CRL-5883; ATCC)
SK-LU-1 (cat. no. HTB-57; ATCC)
ZR-75-1 (cat. no. CRL-1500; ATCC)
HCC70 (cat. no. CRL-2315; ATCC)
MDA-MB-231 (cat. no. HTB-26; ATCC)
U2OS EJ7-GFP and DR-GFP cells were a gift from Dr. Jeremy Stark (City of Hope)

Authentication

The identity of NCI-H838, NCI-H1650, SK-LU-1, ZR-75-1, HCC70 and MDA-MB-231 cell lines was confirmed by STR profiling.

Mycoplasma contamination

All cell lines were frequently examined for mycoplasma contamination using the MycoAlert Mycoplasma Detection Kit (Lonza).

Commonly misidentified lines
(See ICLAC register)

No cell lines were classified as misidentified (ICLAC v12 release).

Plants

Seed stocks	No plant material was used in the study.
Novel plant genotypes	No novel plant genotypes were produced in the study.
Authentication	No plant authentication procedures were included in the study.

Flow Cytometry

Plots

Confirm that:

☒ The axis labels state the marker and fluorochrome used (e.g. CD4-FITC).

☒ The axis scales are clearly visible. Include numbers along axes only for bottom left plot of group (a 'group' is an analysis of identical markers).

☒ All plots are contour plots with outliers or pseudocolor plots.

☒ A numerical value for number of cells or percentage (with statistics) is provided.

Methodology

Sample preparation	*Quantification of apoptosis by flow-cytometry<br>Apoptotic cells were labeled for flow cytometry using a Dead Cell Apoptosis Kit with Annexin V FITC & Propidium Iodide (cat. no. V13242, Invitrogen). Briefly, cells were seeded at a density of 500,000 cells/well in a 6-well plate and treated with 500 ng/mL doxycycline for 72 h. Next, cell pellets were collected and resuspended in 100 µL of 1X annexin-binding buffer containing 5 µL of FITC annexin V antibody and 1 µL of 100 µg/mL propidium iodide (PI) working solution. Following an incubation at room temperature for 15 min, 400 µL of 1X annexin-binding buffer was added to each sample. Stained cells were analyzed by flow cytometry in a BD LSRFortessa cell analyzer (BD Biosciences), measuring the fluorescence emission at 530 nm (FITC) and 575 nm (PI). Cells were gated and classified as live (negative for both annexin V and PI), early apoptotic (positive for annexin V), or late apoptotic (positive for both annexin V and PI). Image quantification was performed in FlowJo v 10.8.1 software.<br><br>*Cell cycle analysis by flow-cytometry<br>Cell cycle analysis was performed by in vitro labeling of cells with the APC BrdU Flow Kit (cat. no. 552598, BD Biosciences). First, 1 million cells were treated with 500 ng/mL doxycycline for 48 h. Next, 10 µM BrdU solution was added to the culture media and cells were incubated for 2 h. Cells were then fixed and permeabilized according to the manufacturer's protocol. Next, samples were treated with 60 µg of DNase and incubated for 1 h at 37 C to expose the incorporated BrdU. Finally, cells were stained for BrdU (1:50 antibody dilution) and total DNA (7-AAD 1:50 solution dilution) and analyzed by flow cytometer in a BD LSRFortessa cell analyzer (BD Biosciences) using 488 nm and 640 nm lasers. Cells were gated and assigned to G1, S, or G2 phases of the cell cycle.
Instrument	BD LSRFortessa cell analyzer (BD Biosciences)
Software	FlowJo v 10.8.1 software.
Cell population abundance	No cell sorting was performed.
Gating strategy	An initial SSC/FSC gate was determined to exclude cell debris and doublets. For apoptotic assays, FITC and PI positivity thresholds were defined by using negative (no stain) and positive (FITC-only, PI-only) control populations.

☒ Tick this box to confirm that a figure exemplifying the gating strategy is provided in the Supplementary Information.
